# Pre‐exposure prophylaxis implementation gaps among people vulnerable to HIV acquisition: a cross‐sectional analysis in two communities in western Kenya, 2021–2023

**DOI:** 10.1002/jia2.26372

**Published:** 2024-11-04

**Authors:** Matthew L. Romo, Glenna Schluck, Josphat Kosgei, Christine Akoth, Rael Bor, Deborah Langat, Curtisha Charles, Paul Adjei, Britt Gayle, Elyse LeeVan, David Chang, Adam Yates, Margaret Yacovone, Julie A. Ake, Fred Sawe, Trevor A. Crowell, for the Multinational Observational Cohort of HIV and other Infections (MOCHI) Study Group

**Affiliations:** ^1^ U.S. Military HIV Research Program CIDR, Walter Reed Army Institute of Research Silver Spring Maryland USA; ^2^ Henry M. Jackson Foundation for the Advancement of Military Medicine Bethesda Maryland USA; ^3^ U.S. Military HIV Research Program Walter Reed Army Institute of Research ‐ Africa Kericho Kenya; ^4^ HJF Medical Research International Kericho Kenya; ^5^ National Cancer Institute, National Institutes of Health Rockville Maryland USA; ^6^ National Institute of Allergy and Infectious Diseases, National Institutes of Health Rockville Maryland USA

**Keywords:** pre‐exposure prophylaxis, HIV, tenofovir, Kenya, implementation science, social determinants of health

## Abstract

**Introduction:**

Despite the increasing availability of prevention tools like pre‐exposure prophylaxis (PrEP), HIV incidence remains disproportionately high in sub‐Saharan Africa. We examined PrEP awareness, uptake and persistence among participants enrolling into an HIV incidence cohort in Kenya.

**Methods:**

We used cross‐sectional enrolment data from the Multinational Observational Cohort of HIV and other Infections (MOCHI) in Homa Bay and Kericho, Kenya. The cohort recruited individuals aged 14–55 years with a recent history of sexually transmitted infection, transactional sex, condomless sex and/or injection drug use. Participants completed questionnaires on PrEP, demographics and sexual behaviours. We used multivariable robust Poisson regression to estimate adjusted prevalence ratios (aPRs) and 95% confidence intervals (CIs) for associations with never hearing of PrEP, never taking PrEP and ever stopping PrEP.

**Results:**

Between 12/2021 and 5/2023, 399 participants attempted the PrEP questionnaire, of whom 316 (79.2%) were female and median age was 22 years (interquartile range 19–24); 316 of 390 participants (81.0%) engaged in sex work or transactional sex. Of 396 participants who responded to the question, 120 (30.3%) had never heard of PrEP. Of 275 participants who had heard of PrEP, 206 (74.9%) had never taken it. Of 69 participants who had ever taken PrEP, 50 (72.5%) stopped it at some time prior to enrolment. Participants aged 15–19 years more often reported never taking PrEP compared with those 25–36 years (aPR 1.31, 95% CI: 1.06–1.61). Participants who knew someone who took PrEP less often reported never hearing about PrEP (aPR 0.10, 95% CI: 0.04–0.23) and never taking PrEP (aPR: 0.69, 95% CI: 0.60–0.80). Stopping PrEP was more common among participants with a weekly household income ≤1000 versus >1000 Kenyan shillings (aPR 1.40, 95% CI: 1.02–1.93) and those using alcohol/drugs before sex (aPR 1.53, 95% CI: 1.03–2.26). Stopping PrEP was less common among those engaging in sex work or transactional sex (aPR 0.6, 95% CI: 0.40–0.92).

**Conclusions:**

We identified substantial gaps in PrEP awareness, uptake and persistence, which were associated with potential system‐ and individual‐level risk factors. Our analyses also highlight the importance of increasing PrEP engagement among individuals who do not know others taking PrEP.

## 1 INTRODUCTION

Kenya has about 1.4 million people living with HIV (4% prevalence) and although the incidence is decreasing, 35,000 people newly acquired HIV in 2021 [1]. HIV epidemiology varies geographically, with nine counties primarily in western Kenya accounting for 57% of all new HIV diagnoses in 2021 [2, 3]. Kenya developed an implementation framework for oral tenofovir‐based pre‐exposure prophylaxis (PrEP) in 2017 [4] and introduced it into guidelines in 2018 [5]. Despite increased PrEP initiation country‐wide [6], progress has been slower than desired [4] and studies have reported low uptake and high rates of discontinuation in some populations [7, 8, 9, 10]. Better understanding of implementation gaps and who they affect may inform ways to enhance PrEP engagement, particularly among “key” and other vulnerable populations underserved by Kenya's HIV response [11]. We assessed PrEP awareness, use and persistence among individuals vulnerable to HIV acquisition in western Kenya and identified variables associated with gaps in PrEP implementation.

## 2 METHODS

### 2.1 Study population

We used enrolment data from the Multinational Observational Cohort of HIV and other Infections (MOCHI; Clinicaltrials.gov NCT05147519), an HIV incidence study in Kericho and Homa Bay, Kenya. Recruitment occurred primarily in bars, entertainment venues, and fish markets [12]. A target sample size of 400 was chosen for 95% confidence of detecting ≥3 cases/100 person‐years (i.e. incidence sufficient to support efficacy testing of HIV prevention interventions) if the true incidence were at least 4.5 cases per 100 person‐years.

Eligible participants were 14–55 years of age, not living with HIV (based on non‐reactive antibody test) and satisfied ≥1 of the following criteria in the previous 24 weeks: (1) documented newly diagnosed bacterial sexually transmitted infection (STI), herpes simplex virus or acute hepatitis C virus; (2) self‐reported sexual intercourse in exchange for money as a regular source of income; (3) self‐reported condomless vaginal or anal intercourse with ≥3 different partners living with HIV or with unknown status; (4) injection drug use; and (5) self‐reported anal/neovaginal intercourse. A positive urine pregnancy test result was exclusionary to mimic the potential participant population in an early‐phase clinical trial.

Informed consent was required for all participants; parent/guardian consent was not required for those aged 15–17 years per local guidelines [13]. The study was approved by institutional review boards at the Kenya Medical Research Institute (#4237) and Walter Reed Army Institute of Research (#2877).

### 2.2 Data collection and measures

Participants completed questionnaires covering demographics, behaviours and PrEP in English or Kiswahili primarily by computer‐assisted self‐interview at the screening and enrolment visits.

Non‐mutually exclusive key populations were defined as follows: people who engaged in sex work or transactional sex (i.e. identifying current occupation as a sex worker or reported transactional sex in the past 12 weeks); males who have sex with males (MSM; i.e. males with a male partner in the past 12 weeks), transgender people (i.e. gender identity other than the sex assigned at birth) and people who inject drugs (i.e. in the past 12 weeks). Inconsistent/no condom use in the past 12 weeks was ascertained by asking about the percentage of sex acts with a condom in separate questions by partner sex, main or casual partner type, sex type (i.e. anal, vaginal, neovaginal) and insertive or receptive role when applicable.

The PrEP questionnaire included questions about PrEP awareness, use and agreement or disagreement with statements related to concerns about PrEP and interest in PrEP options using a 5‐point Likert scale. We defined three implementation gaps related to PrEP awareness and use: (1) never heard of PrEP; (2) never taken PrEP (among those who had heard of it); and (3) ever stopped PrEP (among those who had ever used it). The latter outcome comprised those who reported ever stopping PrEP and those who reported ever taking PrEP but were not taking it at enrolment.

### 2.3 Statistical analyses

Descriptive analyses included frequencies and proportions for all variables. We used multivariable Poisson regression with robust standard errors [14] to compute adjusted prevalence ratios (aPRs) and 95% confidence intervals (CIs) to examine associations between each implementation gap and exposure variables. Analyses were conducted using SAS 9.4 (SAS Institute Inc., Cary, NC). Statistical significance was determined by *p*<0.05.

## 3 RESULTS

### 3.1 Participant characteristics

Between December 2021 and May 2023, 485 individuals were screened for eligibility, 407 were enrolled and 399 who attempted the PrEP questionnaire were included in these analyses. Median age was 22 years (interquartile range [IQR] 19–24) and 316 (79.2%) were female (Table 1). Common occupations included sex worker (260; 65.3%), fisher or fish trader (32; 8.0%) and entertainment/hospitality (32, 8.0%). Most participants could be classified as a key population, with 316 (81.0%) participants who engaged in sex work or transactional sex, 43 (10.9%) MSM, 6 (1.5%) transgender people and 15 (3.8%) participants with injection drug use.

**Table 1:** Study population characteristics and factors associated with never hearing of PrEP, never taking PrEP and ever stopping PrEP

	All participants (*n* = 399)		Never heard of PrEP (*n* = 396)				Never taken PrEP (*n* = 275)				Ever stopped PrEP (*n* = 69)			
	*n*	Column %	*n*	Row %	aPR	95% CI	*n*	Row %	aPR	95% CI	*n*	Row %	aPR	95% CI
**Total**	399	100%	120	30.3	–	–	206	74.9	–	–	50	72.5	–	–
**Sex assigned at birth**														
Male	83	20.8	20	24.1	1.17	0.77, 1.77	46	73.0	1.01	0.82, 1.23	13	76.5	0.94	0.60, 1.45
Female	316	79.2	100	31.6	–	–	160	75.5	–	–	37	71.2	–	–
**Age group** ^a^														
15–19 years	105	26.3	37	35.2	0.95	0.52, 1.73	61	91.0	**1.31**	**1.06, 1.61**	6	100.0	0.96	0.56, 1.67
20–24 years	213	53.4	69	32.4	1.08	0.59, 1.97	103	73.0	1.07	0.86, 1.34	27	71.1	0.87	0.56, 1.36
25–36 years	81	20.3	14	17.3	–	–	42	62.7	–	–	17	68.0	–	–
**Marital or relationship status**														
Married or cohabitating	36	9.0	3	8.3	–	–	28	84.8	–	–	4	80.0	–	–
Not married or cohabitating	362	91.0	116	32	2.28	0.69, 7.52	178	73.6	0.89	0.72, 1.11	46	71.9	0.70	0.38, 1.26
**Years of education**														
<12	209	52.5	73	34.9	0.98	0.73, 1.33	104	78.2	1.04	0.91, 1.20	20	69.0	0.81	0.53, 1.23
≥12	189	47.5	46	24.3	–	–	102	71.8	–	–	30	75.0	–	–
**Weekly household income**														
≤1000 KES^b^	133	33.4	48	36.1	1.22	0.93, 1.59	64	76.2	0.99	0.84, 1.15	18	90.0	**1.40**	**1.02, 1.93**
>1000 KES^b^	265	66.6	71	26.8	–	–	142	74.3	–	–	32	65.3	–	–
**Study site location**														
Kericho	181	45.4	84	46.4	1.45	0.90, 2.34	81	85.3	1.08	0.89, 1.31	12	85.7	1.23	0.74, 2.05
Homa Bay	218	54.6	36	16.5	–	–	125	69.4	–	–	38	69.1	–	–
**Distance from home to study facility**														
≤3 km	96	24.3	14	14.6	–	–	61	75.3	–	–	15	75.0	–	–
>3 km	299	75.7	104	34.8	0.97	0.56, 1.70	143	74.5	0.86	0.73, 1.02	35	71.4	0.97	0.66, 1.42
**Engaged in sex work or transactional sex**														
	316^c^	81.0	103	32.6	0.95	0.55, 1.63	154	73.7	0.89	0.72, 1.09	37	67.3	**0.60**	**0.40, 0.92**
**Males who have sex with males**														
	43^d^	10.9	12	27.9	^j^	^j^	15	48.4	^j^	^j^	12	75.0	^j^	^j^
**Transgender identity**														
	6^e^	1.5	1	16.7	^j^	^j^	3	60.0	^j^	^j^	1	50.0	^j^	^j^
**Injection drug use**														
	15^f^	3.8	3	20.0	^j^	^j^	9	80.0	^j^	^j^	3	100	^j^	^j^
**Partner with HIV or unknown HIV status in the past 12 weeks**														
	279^g^	69.9	99	35.5	1.40	0.81, 2.41	136	76.4	1.14	0.95, 1.36	34	81.0	1.22	0.83, 1.79
**Condom use for anal or (neo)vaginal sex in the past 12 weeks**														
Inconsistent	306	79.9	89	29.1	0.94	0.67, 1.31	159	74.3	0.99	0.83, 1.16	41	74.5	0.80	0.51, 1.27
Consistent	77	20.1	25	32.5	–	–	39	76.5	–	–	8	66.7	–	–
**Alcohol and/or drugs before sex in the past 12 weeks**														
	234	60.3	86	36.8	1.24	0.88, 1.74	113	76.4	1.06	0.90, 1.24	28	80.0	**1.53**	**1.03, 2.26**
**Self‐reported STI in the past 12 weeks**														
	44	11.5	8	18.2	0.75	0.43, 1.32	24	66.7	0.97	0.78, 1.20	10	83.3	1.05	0.69, 1.60
**More than one course of HIV PEP in a lifetime**														
Yes	33	8.9	1	3	^j^	^j^	10	31.3	**0.43**	**0.27, 0.68**	14	63.6	0.89	0.59, 1.34
No, or never heard of PEP	336^h^	91.1	103	30.7	–	–	188	81.7	–	–	32	76.2	–	–
**Knew someone who took PrEP**														
	164	41.2	8^i^	4.9	**0.10**	**0.04–0.23**	95	61.7	**0.69**	**0.60, 0.80**	43	72.9	0.88	0.49, 1.58

*Notes*: This table shows frequencies and column percentages for all study population characteristics; frequencies and row percentages for all three implementation gaps; and results from fully adjusted regression models for never heard of PrEP (*n* = 360), never taken PrEP (*n* = 239) and ever stopped PrEP (*n* = 60). Results for never heard of PrEP were limited to the 396 participants who answered the question about having heard about PrEP among the 399 participants who attempted the questionnaire. Results for never taken PrEP were limited to the 275 participants who answered the question about taking PrEP among the 276 participants who reported ever having heard about PrEP. Results for ever stopped PrEP included all 69 participants who reported taking PrEP, since they also all answered the question about stopping PrEP. For the regression models, statistically significant results are bolded. Missing data were handled using listwise deletion.

Abbreviations: aPR, adjusted prevalence ratio; CI, confidence interval; KES, Kenyan Shillings; PEP, post‐exposure prophylaxis; PrEP, pre‐exposure prophylaxis; STI, sexually transmitted infection.

^a^ Although individuals aged 14–55 years were eligible, only participants aged 15–36 years enrolled.

^b^ 1000 KES was equivalent to approximately 7 USD at the time of the survey and was the 20th percentile of responses.

^c^ *n* = 260 reported their current occupation as “sex worker.”

^d^ Among 43 males who have sex with males, *n* = 19 also reported engaging in sex work/transactional sex and *n* = 2 also reported injection drug use.

^e^ Among six transgender participants, *n* = 3 were transgender women and *n* = 3 were transgender men; *n* = 2 also reported sex work/transactional sex and *n* = 2 reported engaging in both sex work/transactional sex and injection drug use.

^f^ Among 15 participants with injection drug use, *n* = 10 also reported engaging in sex work/transactional sex.

^g^ *n* = 12 participants had partners known to be living with HIV.

^h^ *n* = 114 had never heard of PEP; *n* = 184 had heard of PEP but had never taken it; *n* = 38 had taken one course of PEP.

^i^ Likely represents individuals who recalled knowing someone who took PrEP after being provided with information on PrEP during the survey.

^j^ Not included in the multivariable model because of insufficient sample size and/or collinearity.

### 3.2 PrEP awareness and use, and implementation gaps

**Figure 1:**
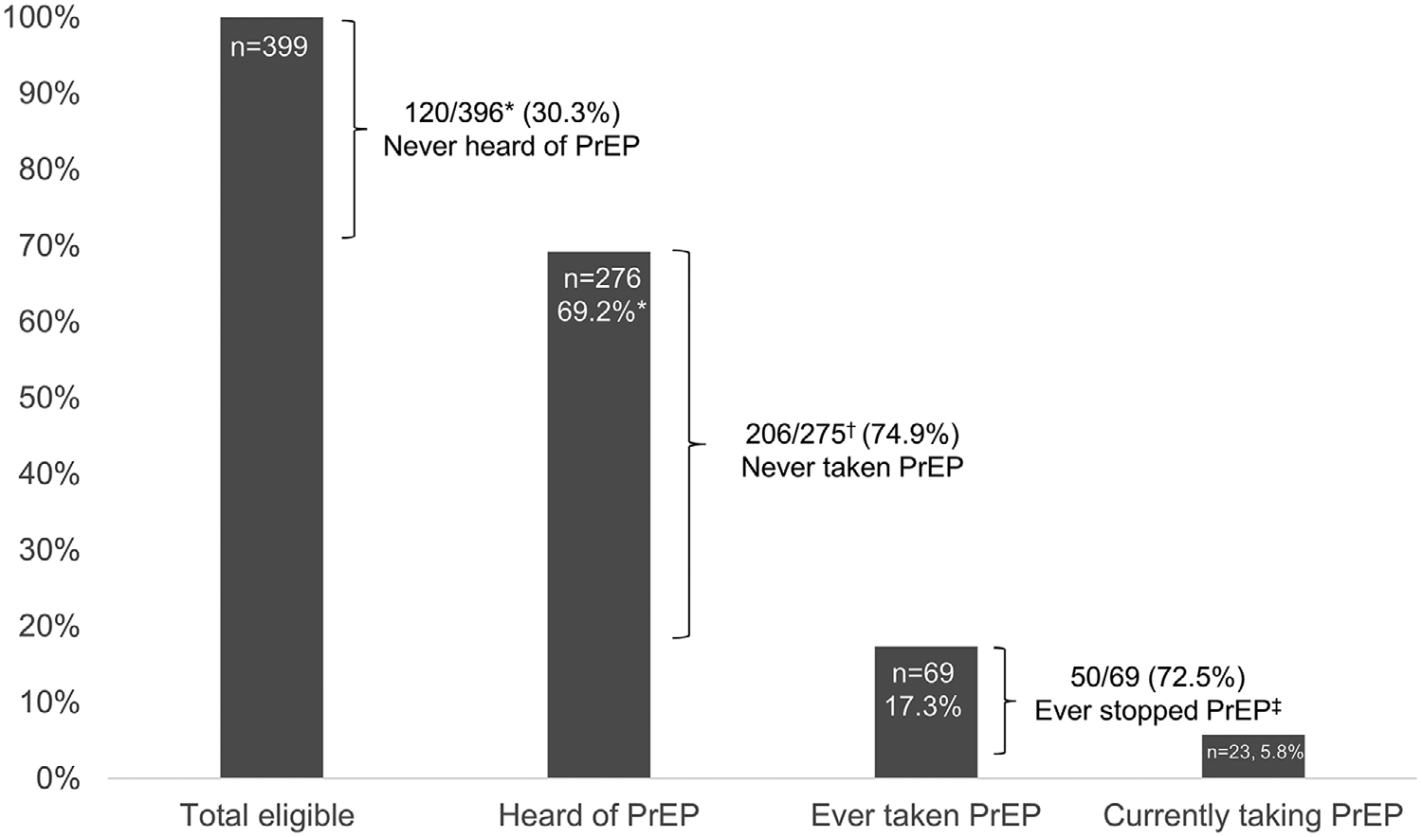
PrEP eligibility, awareness and use among 399 participants vulnerable to HIV acquisition. PrEP, pre‐exposure prophylaxis. *Three participants eligible for PrEP did not respond to the question about hearing of PrEP, so the denominator is 396. ^†^One participant who had heard of PrEP did not respond to the question about ever taking PrEP, so the denominator is 275. ^‡^Inclusive of participants who reported ever stopping PrEP and participants who reported ever taking PrEP but were not currently taking it. Therefore, the bracket designating having ever stopped PrEP extends beyond the bar for currently taking PrEP. This figure shows the PrEP engagement cascade, starting at the left with the total number eligible for PrEP, the proportion who had heard of PrEP, the proportion who had ever taken PrEP and the proportion who were currently taking PrEP. Between the bar graphs, we show three gaps in PrEP implementation: never hearing of PrEP, never taking PrEP and ever stopping PrEP. PrEP awareness and use among key populations: Among the 316 people who engaged in sex work or transactional sex, 210 (66.5%) had heard of PrEP, 55 (17.4%) had ever taken PrEP and 21 (6.6%) were currently taking PrEP. Among the 43 MSM, 31 (72.1%) had heard of PrEP, 16 (37.2%) had ever taken PrEP and 5 (11.6%) were currently taking PrEP. Among the six transgender people, five (83.3%) heard of PrEP, two (33.3%) had ever taken PrEP and one (16.7%) was currently taking PrEP. Among the 15 people with injection drug use, 12 (80.0%) had heard of PrEP, 3 (20.0%) had ever taken PrEP and 1 (6.7%) was currently taking PrEP.

Of 399 participants, 276 (69.2%) had heard of PrEP, 69 (17.3%) had ever taken PrEP and 23 (5.8%) were currently taking PrEP (Figure 1). Of 396 who answered the question about hearing of PrEP, 120 (30.3%) had never heard of PrEP. Of 275 participants who reported having heard of PrEP, 206 (74.9%) had never taken PrEP. Of 69 participants who had ever taken PrEP, 50 (72.5%) had ever stopped taking it.

Among the 23 participants currently taking PrEP, median duration of PrEP use was 6 months (IQR 2–10) and 16 (69.6%) reported 100% adherence in the past week. Most were accessing PrEP at a government clinic (12 [52.2%]) or a community‐based organization (6 [26.1%]) and reported not paying for it (21 [91.3%]). Condom use was reported to be more frequent after using PrEP among 14 (60.9%) participants. One participant reported problems accessing PrEP, related to its availability (e.g. stock outs).

Among the 50 participants who stopped PrEP, eight (17.4%) reported problems accessing PrEP, including problems related to its availability (4 [57.1%]) or being unable to travel to pick it up (3 [42.9%]). The main reasons for stopping PrEP were trusting their partner (11 [22.0%]), unable to access (5 [10.0%]), side effects (5 [10.0%]) and not wanting others to know (4 [8.0%]).

### 3.3 Concerns about PrEP and interest in PrEP options

**Figure 2:**
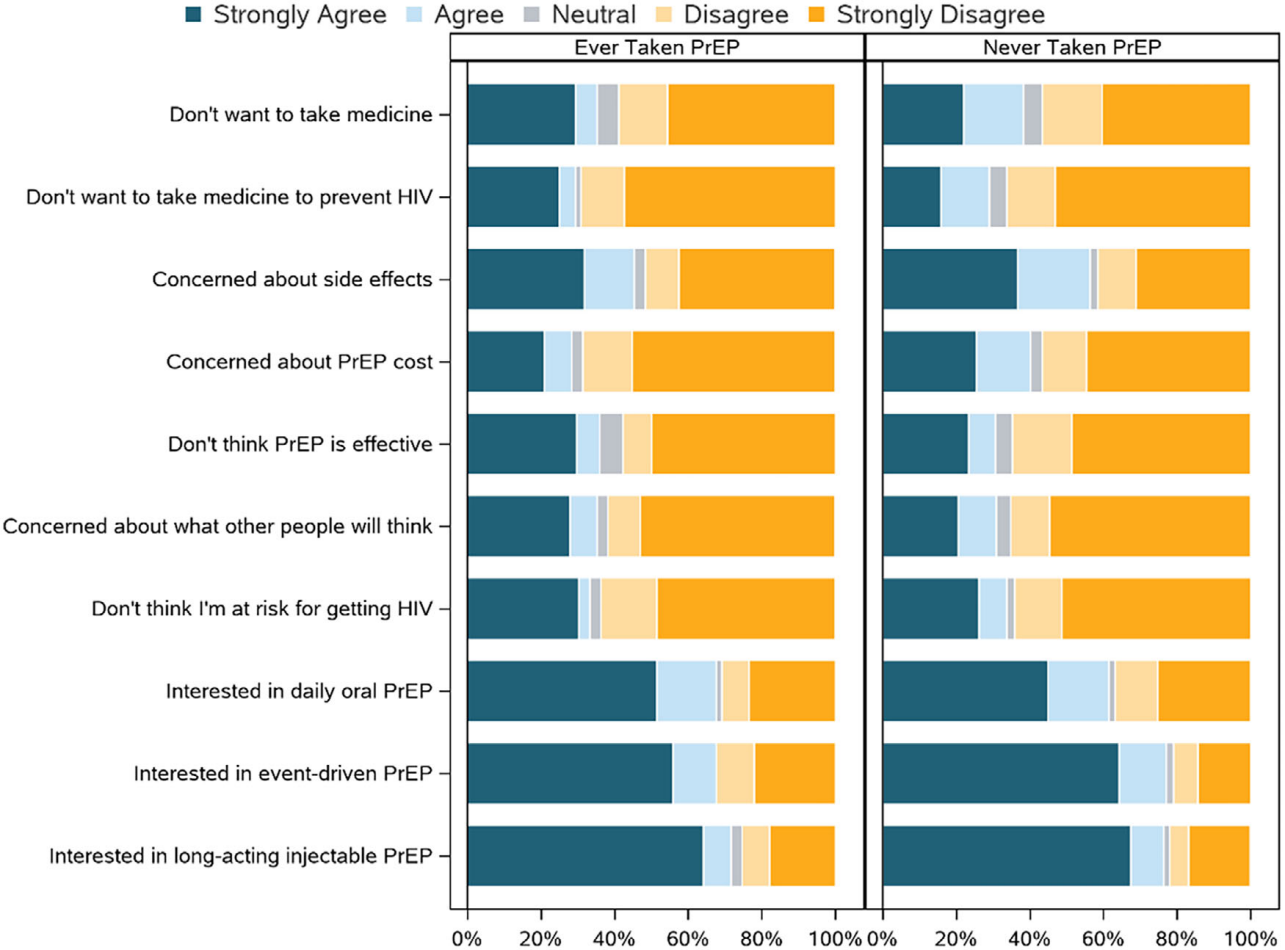
Concerns about PrEP and interest in PrEP options stratified by ever having ever taken PrEP. PrEP, pre‐exposure prophylaxis. This figure shows responses to agreement/disagreement (based on a 5‐point Likert scale) to statements about concerns about PrEP and interest in PrEP options, stratified by ever taken PrEP (left) and never taken PrEP (right).

The most predominant concern among those who had ever taken PrEP and never taken PrEP was side effects (agree/strongly agree, respectively: 30/66 [45.5%], 163/289 [56.4%]), followed by doubts about PrEP efficacy for those who had ever taken PrEP (agree/strongly agree: 23/64 [35.9%]) and cost for those who had never taken PrEP (agree/strongly agree: 101/251 [40.2%]; Figure 2).

Among those who had ever taken PrEP, the strongest interest was for long‐acting injectable PrEP (agree/strongly agree: 48/67 [71.6%]), with the same level of interest for event‐driven PrEP and daily oral PrEP (agree/strongly agree: 46/68 [67.6%], for both options). For those who had never taken PrEP, interest was similarly high for event‐driven PrEP (agree/strongly agree: 235/305 [77.0%]) and long‐acting injectable PrEP (agree/strongly agree: 233/305 [76.4%]), followed by daily oral PrEP (agree/strongly agree: 185/301 [61.5%]).

### 3.4 Factors associated with never hearing about PrEP, never taking PrEP and ever stopping PrEP

In multivariable models (Table 1), never hearing about PrEP was significantly less common among those who reported knowing someone who took PrEP (aPR 0.10, 95% CI: 0.04–0.23). Never taking PrEP was significantly less common among those who reported having ever taken more than one course of HIV post‐exposure prophylaxis (aPR 0.43, 95% CI: 0.27–0.68) and among those who reported knowing someone who took PrEP (aPR 0.69, 95% CI: 0.6–0.8). Never taking PrEP was significantly more common among participants aged 15–19 years compared with those 25–36 years (aPR 1.31, 95% CI: 1.06–1.61). Ever stopping PrEP was significantly more common among those with a weekly household income ≤1000 versus >1000 Kenyan shillings (aPR 1.40, 95% CI: 1.02–1.93) and those who reported using alcohol and/or drugs before sex versus not (aPR 1.53, 95% CI: 1.03–2.26). Ever stopping PrEP was less common among those who engaged in sex work or transactional sex (aPR 0.60, 95% CI: 0.40–0.92).

## 4 DISCUSSION

Despite the representation of groups with disproportionately high HIV incidence, PrEP awareness, uptake and persistence were low, corroborating other reports from Kenya and other African countries [7, 8, 9, 10, 15, 16, 17]. Consistent with Kenya's initial tiered rollout of PrEP by county‐level HIV burden [4], we found numerically greater implementation gaps in Kericho versus Homa Bay, which are in low‐ and high‐incidence counties, respectively, but these differences were not statistically significant in multivariable modelling. Kenya's 2022 implementation framework for PrEP included an explicit focus on key populations, in addition to targeting high‐incidence counties [3]. The gaps in PrEP engagement identified among our study population support this additional focus, as key populations are vulnerable to HIV acquisition wherever they reside and should be prioritized for existing and novel HIV prevention tools. Our findings also show the importance of increasing PrEP engagement among the youngest age subgroup (i.e. 15–19 years old) who least often accessed PrEP.

Knowing someone who took PrEP was associated with greater PrEP awareness and uptake, highlighting the need to increase PrEP engagement among people who do not have PrEP users in their social networks. Key populations face pervasive stigma and discrimination in healthcare settings [18, 19, 20], yet some like female sex workers have high levels of social connectivity that could be leveraged for HIV prevention efforts [21]. A modification of the Social‐Ecological Model to HIV prevention emphasizes how both an individual's community and interpersonal networks can influence HIV risk and be incorporated into interventions [22]. Implementation strategies that include peers for referral, navigation and/or delivery of PrEP services have the potential to increase PrEP engagement [23, 24, 25] and are recommended by the World Health Organization [26]. More evidence is needed for specific approaches that could be integrated into existing HIV programming.

Stopping PrEP was common and associated with lower income and alcohol/drug use before sex. In the literature, associations for these variables with PrEP discontinuation have been inconsistent [27, 28]. Programmes that provide PrEP services should target individuals experiencing system‐ and individual‐level risk factors with additional monitoring and resources, as they are at greater risk for adverse PrEP outcomes, such as in PEPFAR's Determined, Resilient, Empowered, AIDS‐Free, Mentored and Safe (DREAMS) partnership [29]. Participants who engaged in sex work or transactional sex less often reported to have stopped PrEP, which might reflect a greater perceived ongoing need for PrEP relative to other groups. Perceived HIV risk has been robustly associated with PrEP persistence [30, 31].

The low uptake of PrEP, high prevalence of stopping PrEP, varying concerns about PrEP and interest in different PrEP options highlight the importance of offering PrEP options that meet people's needs. For some, long‐acting injectable PrEP is preferred over oral [32, 33, 34, 35] and its implementation is anticipated to reduce HIV incidence in African countries [36]. However, PrEP modalities that meet other needs, for example dual pregnancy prevention [37], and novel service delivery models, for example through community pharmacies [38, 39], have the potential to increase PrEP engagement. Side effects were a predominant concern, especially among people who had never taken PrEP before, which highlights the importance of education and addressing tolerability. Out‐of‐pocket costs were also a predominant concern among those who had never taken PrEP before; thus, direct and indirect costs are possible barriers to address.

Regarding limitations, our questionnaire did not ask specifically about event‐driven PrEP use, which was not in local guidelines when the study was designed [5]. Future surveys should evaluate event‐driven PrEP in Kenya and identify ways to optimize its use for eligible populations. Our sampling strategy was non‐probabilistic and thus precluded the generation of representative estimates; however, it allowed for the effective recruitment of populations vulnerable to HIV. Some participants from key populations may have been misclassified because our definitions were based on questions with 12‐week recall. We had few participants who were transgender or who injected drugs, precluding a robust assessment of PrEP engagement in these populations.

## 5 CONCLUSIONS

We identified substantial gaps in PrEP awareness, uptake and persistence in this cohort of people vulnerable to HIV acquisition in Kenya and these gaps were associated with potential system‐ and individual‐level risk factors. Our analyses also highlight the importance of increasing PrEP engagement among individuals who do not know others taking PrEP.

## COMPETING INTERESTS

The authors declare that they have no competing interests.

## AUTHORS’ CONTRIBUTIONS

MLR, GS, JK, BG and TAC conceived and designed the analysis with critical input at various stages from CC, PA, EL, DC, MY, JAA and FS. CA, RB and DL collected and managed data. GS and AY conducted the statistical analysis. MLR wrote the first draft of the manuscript. GS, JK, CA, RB, DL, CC, PA, BG, EL, DC, AY, MY, JAA, FS and TAC edited and provided input for the manuscript, and approved the final version.

## FUNDING

This work was supported by a cooperative agreement (W81XWH‐18‐2‐0040) between the Henry M. Jackson Foundation for the Advancement of Military Medicine, Inc., and the U.S. Department of Defense (DoD). This research was funded, in part, by the U.S. National Institute of Allergy and Infectious Diseases (AAI20052001). The investigators have adhered to the policies for protection of human research participants as prescribed in AR 70–25.

## DISCLAIMER

This material has been reviewed by the Walter Reed Army Institute of Research. There is no objection to its presentation and/or publication. The opinions or assertions contained herein are the private views of the author, and are not to be construed as official, or as reflecting true views of the Department of the Army or the Department of Defense.

## Data Availability

The data that support the findings of this study are available on request from the corresponding author. The data are not publicly available due to privacy or ethical restrictions.
